# The Spectrum of the Deficiency of Adenosine Deaminase 2: An Observational Analysis of a 60 Patient Cohort

**DOI:** 10.3389/fimmu.2021.811473

**Published:** 2022-01-10

**Authors:** Karyl S. Barron, Ivona Aksentijevich, Natalie T. Deuitch, Deborah L. Stone, Patrycja Hoffmann, Ryan Videgar-Laird, Ariane Soldatos, Jenna Bergerson, Camilo Toro, Cornelia Cudrici, Michele Nehrebecky, Tina Romeo, Anne Jones, Manfred Boehm, Jennifer A. Kanakry, Dimana Dimitrova, Katherine R. Calvo, Hawwa Alao, Devika Kapuria, Gil Ben-Yakov, Dominique C. Pichard, Londa Hathaway, Alessandra Brofferio, Elisa McRae, Natalia Sampaio Moura, Oskar Schnappauf, Sofia Rosenzweig, Theo Heller, Edward W. Cowen, Daniel L. Kastner, Amanda K. Ombrello

**Affiliations:** ^1^ National Institute of Allergy and Infectious Diseases, National Institutes of Health (NIH), Bethesda, MD, United States; ^2^ National Human Genome Research Institute, National Institutes of Health (NIH), Bethesda, MD, United States; ^3^ National Institute of Neurological Diseases and Strokes, National Institutes of Health (NIH), Bethesda, MD, United States; ^4^ Undiagnosed Disease Program, National Institutes of Health (NIH), Bethesda, MD, United States; ^5^ National Heart, Lung, and Blood Institute, National Institutes of Health (NIH), Bethesda, MD, United States; ^6^ National Cancer Institute, National Institutes of Health (NIH), Bethesda, MD, United States; ^7^ Department of Laboratory Medicine, Clinical Center, National Institutes of Health (NIH), Bethesda, MD, United States; ^8^ National Institute of Digestive Diseases and Kidney Diseases, National Institutes of Health (NIH), Bethesda, MD, United States; ^9^ National Institute of Arthritis and Musculoskeletal and Skin Diseases, National Institutes of Health (NIH), Bethesda, MD, United States

**Keywords:** deficiency of adenosine deaminase 2 (DADA2), ADA2, lacunar strokes, immune dysregulation, hematopoietic cell transplantation (HCT), vasculopathy, bone marrow failure, anti-TNF therapy

## Abstract

The deficiency of adenosine deaminase 2 (DADA2) is an autosomal recessively inherited disease that has undergone extensive phenotypic expansion since being first described in patients with fevers, recurrent strokes, livedo racemosa, and polyarteritis nodosa in 2014. It is now recognized that patients may develop multisystem disease that spans multiple medical subspecialties. Here, we describe the findings from a large single center longitudinal cohort of 60 patients, the broad phenotypic presentation, as well as highlight the cohort’s experience with hematopoietic cell transplantation and COVID-19. Disease manifestations could be separated into three major phenotypes: inflammatory/vascular, immune dysregulatory, and hematologic, however, most patients presented with significant overlap between these three phenotype groups. The cardinal features of the inflammatory/vascular group included cutaneous manifestations and stroke. Evidence of immune dysregulation was commonly observed, including hypogammaglobulinemia, absent to low class-switched memory B cells, and inadequate response to vaccination. Despite these findings, infectious complications were exceedingly rare in this cohort. Hematologic findings including pure red cell aplasia (PRCA), immune-mediated neutropenia, and pancytopenia were observed in half of patients. We significantly extended our experience using anti-TNF agents, with no strokes observed in 2026 patient months on TNF inhibitors. Meanwhile, hematologic and immune features had a more varied response to anti-TNF therapy. Six patients received a total of 10 allogeneic hematopoietic cell transplant (HCT) procedures, with secondary graft failure necessitating repeat HCTs in three patients, as well as unplanned donor cell infusions to avoid graft rejection. All transplanted patients had been on anti-TNF agents prior to HCT and received varying degrees of reduced-intensity or non-myeloablative conditioning. All transplanted patients are still alive and have discontinued anti-TNF therapy. The long-term follow up afforded by this large single-center study underscores the clinical heterogeneity of DADA2 and the potential for phenotypes to evolve in any individual patient.

## Introduction

The deficiency of adenosine deaminase 2 (DADA2) was initially described as a syndrome of small and medium-sized vessel vasculitis/vasculopathy manifesting as recurrent episodes of fever, early-onset lacunar strokes, and cutaneous involvement including livedo racemosa, Raynaud’s phenomenon, and polyarteritis nodosa. DADA2 is caused by biallelic loss of function mutations in the *ADA2* gene (formerly *CECR1*) (1, 2).

ADA2 is highly expressed in immune cells and in particular, cells of myeloid lineage. Although the function of ADA2 is poorly characterized, studies in a zebrafish model suggest that ADA2 has a role in the development of hematopoietic cells and maintenance of vascular integrity, which may explain some of the manifestations that have been observed in both the bone marrow and vasculature (1). Deficiency of ADA2 has been associated with polarization of myeloid cells towards M1 (pro-inflammatory) relative to M2 (anti-inflammatory) macrophages. These tissue resident M1 macrophages can produce proinflammatory cytokines and cause a hyperinflammatory environment that is damaging to blood vessels and may contribute to the propensity of affected individuals to develop strokes. Immunohistochemical studies of DADA2 patient skin biopsies have demonstrated endothelial damage and perivascular inflammation with deposition of tumor necrosis factor (TNF), which appears to be resolved with anti-TNF therapy (1, 3). Neutrophils also play an important role in the pathogenesis of DADA2, with reports of circulating low-density granulocytes (LDGs) prone to spontaneous neutrophil extracellular trap (NET) formation (4–6). ADA2 has also been reported to function as a sensor for extracellular purine nucleosides and lack of ADA2 can trigger a type I interferon (IFN) cellular response (7). IFN-driven signaling further promotes immune cell activation and drives additional inflammatory cytokines, including TNF. Upregulation of type II interferon pathway-related genes has also been described in DADA2, suggesting that type II IFN signaling may contribute to DADA2 pathogenesis (3, 8).

In this paper we describe the findings of a large single center longitudinal cohort and expand the phenotypic presentation of the patients to include evidence for lymphocyte driven immune-mediated neutropenia, as well as describe the spectrum of presentations of peripheral vasculopathy. We confirm and extend the previous findings of anti-TNF therapy to include more patient months and long term follow up, as well as delineate circumstances where it is not as effective. We also report the results of hematopoietic cell transplant (HCT) in 6 patients from this cohort. Additionally, this paper emphasizes the importance of offering genetic testing to the siblings of DADA2 patients as there were 7 identified siblings with previously unrecognized clinical manifestations of DADA2 but, upon evaluation, had findings of DADA2 (splenomegaly, immunoglobulin deficiency, elevated acute phase reactants).

The objective of this paper is to describe the findings of a large, single-center cohort of patients with DADA2.

## Methods

### Patients

Fifty-eight consecutive patients with DADA2 were enrolled in a natural history protocol approved by the National Institutes of Health Institutional Review Board and underwent full evaluation at the NIH Clinical Center. Informed consent/assent was obtained as appropriate for all patients and parents. A comprehensive history and physical exam were undertaken to assess the clinical manifestations of DADA2 in every patient and detailed family histories were taken to assess for other potential affected family members.

The patients were evaluated by a core group of consultants (rheumatology, neurology, immunology, dermatology, hepatology, and ophthalmology) at their initial visit, with additional consultation (which could include hematology, nephrology, cardiology, rehabilitation and/or neuropsychology) if needed. The consultant group was individualized for the patients at return visits. All 58 patients underwent at least one exam with magnetic resonance imaging (MRI) of the brain, abdominal ultrasound to assess the abdominal vasculature as well as the liver, spleen, and kidneys, and transient elastography of the liver (Fibroscan). Patients also underwent magnetic resonance angiography (MRA) of the brain, abdomen, or extremities as clinically indicated. Biopsies of skin and/or liver and/or bone marrow were obtained when clinically indicated. Laboratory studies were performed at each visit to assess for inflammation and other organ-specific dysfunction, as well as annual QuantiFERON testing. Unless there were extenuating circumstances, patients were seen approximately 6 months after the initial visit, and then annually.

Two additional patients (numbers 59 and 60) were subsequently identified but did not receive the same comprehensive evaluation as the other patients. Patient 59 was molecularly diagnosed with DADA2 5 years after receiving an HCT for clinically suspected GATA2 deficiency (9). Her data were included in the results of transplantation but not in the clinical and laboratory information. Patient 60, who presented with a history of fevers, lymphadenopathy, hepatosplenomegaly and immune-mediated cytopenias and was suspected to have autoimmune lymphoproliferative syndrome (ALPS), was posthumously identified to have 2 pathogenic mutations in *ADA2* (R169Q/R501X).

### Genetic and Functional Analysis

Genetic testing was done by a combination of standard Sanger sequencing of the 9 coding exons of *ADA2* (NM 001282225.2) and a Multiplex Ligation-dependent Probe Amplification Analysis (MLPA) assay (10). The MLPA assay was used to screen for copy number variation (CNV) in the *ADA2* gene locus. MLPA was performed using a custom-made probe mix from MRC, Holland (Amsterdam, The Netherlands). The procedures were carried out according to the manufacturer’s recommendations and 50 ng of DNA was used for the assay.

### ADA2 Enzyme Activity Assay

To confirm the pathogenicity of new ADA2 variants an Adenosine Deaminase Assay (Diazyme Laboratories) was used to measure ADA2 enzyme activity in patients’ serum as previously described (1) and/or sent to a CLIA certified laboratory.

### Statistical Analysis

Analysis of the effect of anti-TNF treatment on the incidence of stroke was evaluated by comparing the total number of strokes in the cumulative patient-months prior to initiation of anti-TNF therapy with the number of strokes in the cumulative months since starting anti-TNF therapy, using the Exact McNemar test.

Analysis of the effect of anti-TNF therapy on laboratory parameters was evaluated by paired T-test.

## Results

### Genetic and Functional Testing

All 60 patients had biallelic loss of function mutations in *ADA2*, most of which were inherited as compound heterozygous variants. All variants had sufficient evidence for classification as pathogenic or likely pathogenic as defined by the ACMG criteria (**Supplemental Table 1**). When available, serum ADA2 activity was measured in comparison to age-matched controls to provide evidence for variant pathogenicity (ACMG Criterion PS3) (3, 11–13).

Small genomic deletions were found in 4 patients in combination with another pathogenic missense variant. Three individuals from one family were found to have homozygous duplications of exon 7. Four patients had the known splice site mutation c.973-2A>G and 3 siblings from another family carried a splice site variant at c.-47+2T>C all in combination with another variant (10).

### Clinical Manifestations

Fifty-eight patients underwent detailed clinical evaluation to summarize their clinical features. Of these, there was approximately equal gender distribution, 33 were females (57%). This cohort included 8 sibling pairs and 2 families with 3 affected individuals. Clinical features were highly variable in type and severity, yet all individuals with biallelic variants had clinical and/or serological manifestations of DADA2 upon detailed evaluation. Phenotypic variability was noted among affected family members. The average age at presentation to the NIH was 15.95 years (range 0.75 – 64 years). Due to the retrospective diagnosis in several patients with established disease, the average age of onset could not be determined. The mean follow-up of this cohort at the NIH was 5.04 years (1.58-17.35 years) excluding 3 patients who required prolonged hospitalization following HCT. The mean number of NIH clinic visits was 4.21 (1–18). The clinical and laboratory features of the patients are summarized in **Table 1**.

**Table 1:** Summary of clinical and laboratory features.

	Number	%
**GENERAL FEATURES**		
Females	33/58	57%
History of recurrent fever	40/58	69%
**VASCULITIS / VASCULOPATHY**		
**Cutaneous Involvement**		
History of skin involvement	52/58	90%
Livedo racemosa	43/58	74%
PAN/nodules	33/58	57%
Raynaud’s phenomenon	13/58	22%
Ulcerating lesions	3/58	5%
Warts	11/58	19%
**Central Nervous System Involvement**		
Strokes		
Number of patients with strokes	25/58	43%
Ischemic strokes	24/58	41%
Hemorrhagic strokes	7/58	12%
Mean number of strokes per patient	3 (1–11)	
Average age at first stroke	5.7 years (0.4-20)	
Other		
Ptosis (CN III palsy)	12/58	21%
Optic nerve or retinal involvment	9/58	16%
Sensorineural hearing loss	5/58	9%
Transverse myelitis	1/58	2%
**Other Major Organ Involvment**		
Hepatomegaly	29/58	50%
Splenomegaly	31/58	53%
Hepatosplenomegaly	22/58	38%
Elevated hepatic transient elastography	14/56	25%
Portal hypertension	7/58	12%
Renal cortical lesions	13/58	22%
Splenic infarcts	4/58	7%
Systemic hypertension	15/58	26%
Cardiomyopathy	2/58	3%
**HEMATOLOGIC/IMMUNE ABNORMALITIES**		
Diffuse adenopathy	11/58	19%
**Quantitative serum immunoglobulins**		
Abnormal	38/58	66%
Low IgG	32/58	55%
Low IgM	36/58	62%
Low IgA	25/58	43%
Received prolonged immunologlobulin replacement	13/58	22%
**Lymphocyte phenotyping**		
Abnormal	41/51	80%
Low class-switched memory B cells	32/47	68%
Low memory T cells	26/48	54%
Low NK cells	24/51	47%
**Specific antibody testing**		
Inadequate	16/39	41%
**Complete Cell Counts**		
Cytopenia	28/58	48%
Pancytopenia	6/58	10%
Severe anemia	7/58	12%
Immune mediated neutropenia	9/58	16%
Lymphopenia	8/58	14%
Thrombocytopenia	5/58	9%
**Inflammatory markers**		
Elevated sedimentation rate	40/56	71%
Elevated C-reactive protein	47/56	84%
Low serum iron	24/52	46%
**Autoantibodies**		
Positive ANA	16/54	30%
Positive ANCA	2/31	6%

**Figure 1:**
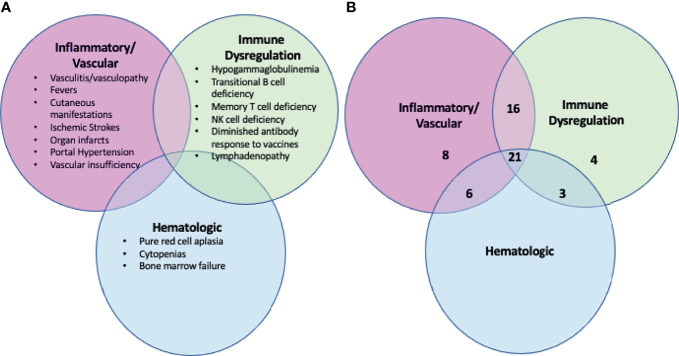
The clinical manifestations of DADA2 are broad and not exclusive. **(A)** Summary of the clinical manifestations of DADA2, including inflammatory, immune dysregulation and hematologic abnormalities. Patients with DADA2 may exhibit any combination of these abnormalities. **(B)** Breakdown by phenotype of the 58 individuals enrolled in the NIH cohort. The numbers reflect the number of individuals in the NIH 58 patient cohort who fall into each phenotypic zone. Forty-six of the 58 individuals have clinical manifestations that span more than one of the broader classes (inflammatory, immune dysregulation, hematologic).

A history of periodic, non-infectious fever was present in 40/58 (69%) of patients. Overall, the clinical manifestations can be grouped under 3 categories: inflammatory (vasculitis/vasculopathy/rash), immune dysregulatory and hematologic abnormalities. Although patients often presented with primary features of one of the categories, after a full evaluation was undertaken, considerable overlapping of the subgroups was observed as shown in **Figure 1**. Notably, whereas there were 8 and 4 patients, respectively, who solely fit in the inflammatory and immune dysregulatory categories, there were no patients who only fit into the hematologic category. Overall, this cohort is skewed towards the inflammatory phenotype, possibly due to the nature of referral.

#### Vasculitis/Vasculopathy

##### Cutaneous Involvement

**Figure 2:**
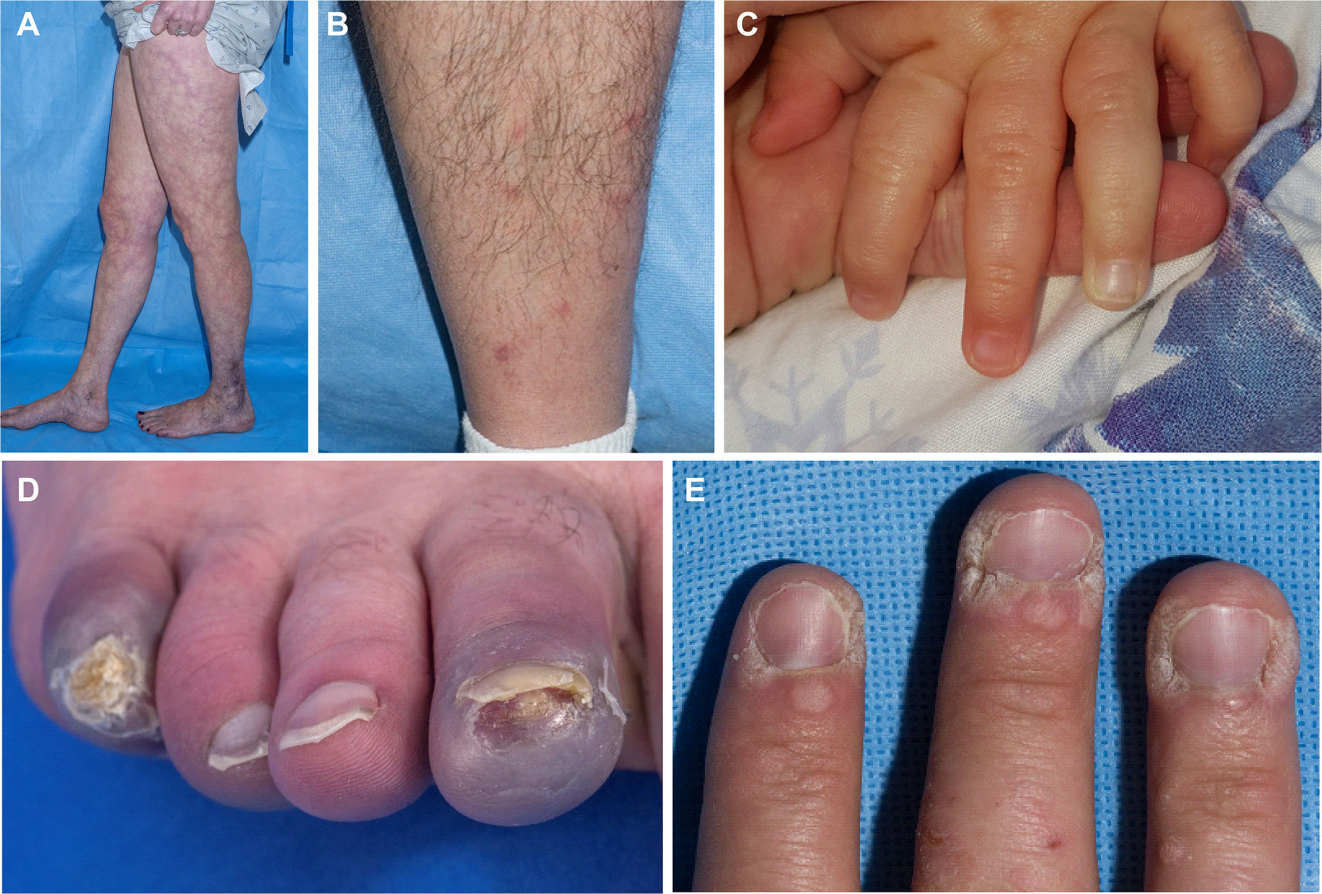
The cutaneous manifestations of DADA2. **(A)** Livedo racemosa on the legs of a 38-year-old. **(B)** Nodules that when biopsied revealed medium vessel vasculitis in a 58-year-old. (Cutaneous polyarteritis nodosa [PAN]). **(C)** Raynaud’s phenomenon in a 13-month-old. **(D)** Acral cyanosis and cutaneous ulcer in a 27-year-old. **(E)** Verruca vulgaris in a 29-year-old.

**Figure 3:**
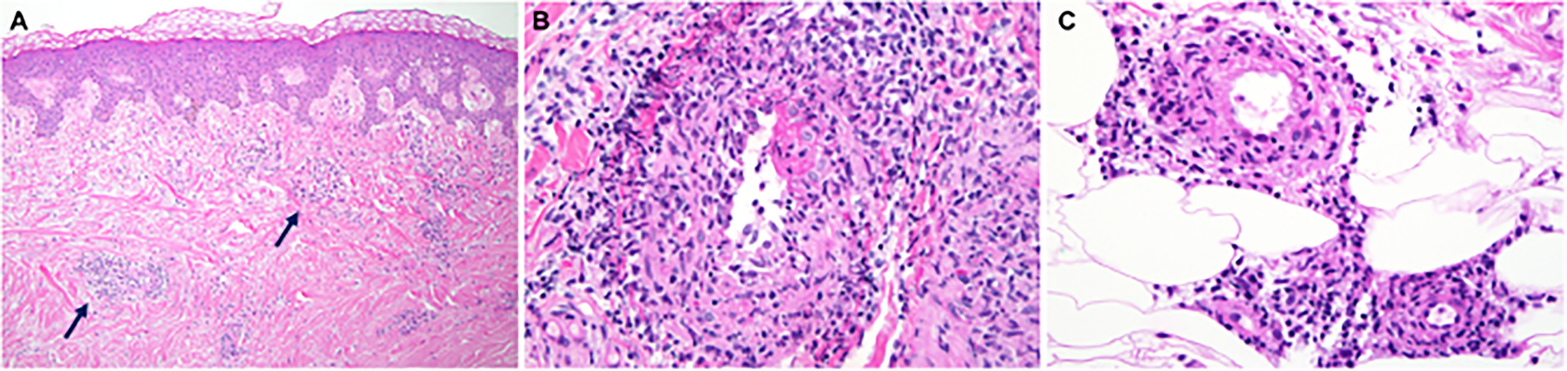
The medium vessel vasculitis in DADA2 patients resembles the vasculitis in polyarteritis nodosa. **(A)** Sections of skin biopsy showing lymphovascular occlusion in medium vessels in the medium-to-deep dermis with inflammatory infiltrate with neutrophils and intravascular thrombi. **(B)** Vasculitis of a medium sized artery characterized by dense transmural infiltrate of mononuclear cells and neutrophils with karyorrhexis, fibrin deposits in vascular lumen, destruction of vessel walls and dense chronic inflammation surrounding the affected blood vessel. **(C)** Vasculitis involving smaller blood vessels of the superficial subcutis. This biopsy was interpreted as possible polyarteritis nodosa.

Skin involvement was the most common manifestation seen in this cohort and was observed in 52/58 patients (90%), with livedo racemosa present in 43/58 patients (74%) (**Figure 2A**). A history of cutaneous features resembling polyarteritis nodosa (PAN)/nodules was noted in 33/58 patients (57%) (**Figure 2B**) and Raynaud’s phenomena was seen in 13/58 patients (22%) (**Figure 2C**). Skin ulcerations were found in 3 patients (**Figure 2D**). Verrucae vulgaris (warts) were seen in 11/58 patients (19%) (**Figure 2E**), likely reflecting underlying immune deficiency (14). There was a range of skin biopsy findings including lesions showing lymphovascular occlusion in medium vessels with inflammatory infiltrates and intravascular thrombi (**Figure 3A**). Vasculitis of medium-sized arterial vessels that resembled PAN was noted with features of mononuclear cells and neutrophils, karyorrhexis, fibrin deposits in vascular lumens, destruction of vessel walls, and dense chronic inflammation surrounding affected blood vessels (**Figure 3B**). Vasculitis involving smaller blood vessels of the superficial cutis was also seen (**Figure 3C**).

##### Central Nervous System Involvement

**Figure 4:**
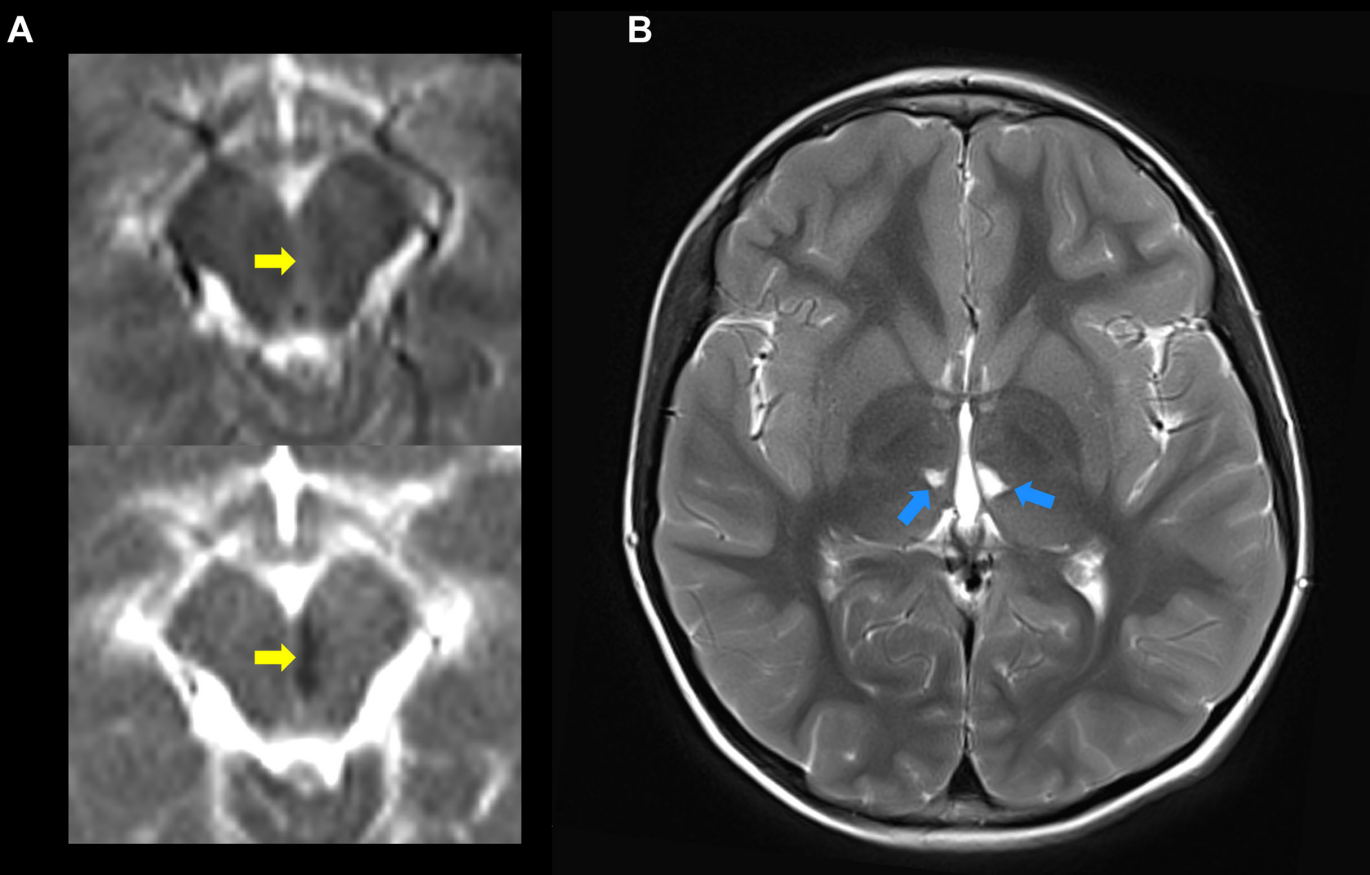
The ischemic strokes in DADA2 may present at an early age and can occur, repeatedly, deep in the brain. **(A)** Shows the diffusion weighted imaging (DWI) (top) and apparent diffusion coefficient (ADC) (bottom) indicative of acute diffusion restriction (yellow arrows) consistent with a new left-paramedial midbrain infarct. **(B)** The same study, on T2 images a few cuts above, shows evidence of two prior lacunar strokes on the thalamus (blue arrows). These strokes occurred in a 3 year 11-month-old boy.

**Figure 5:**
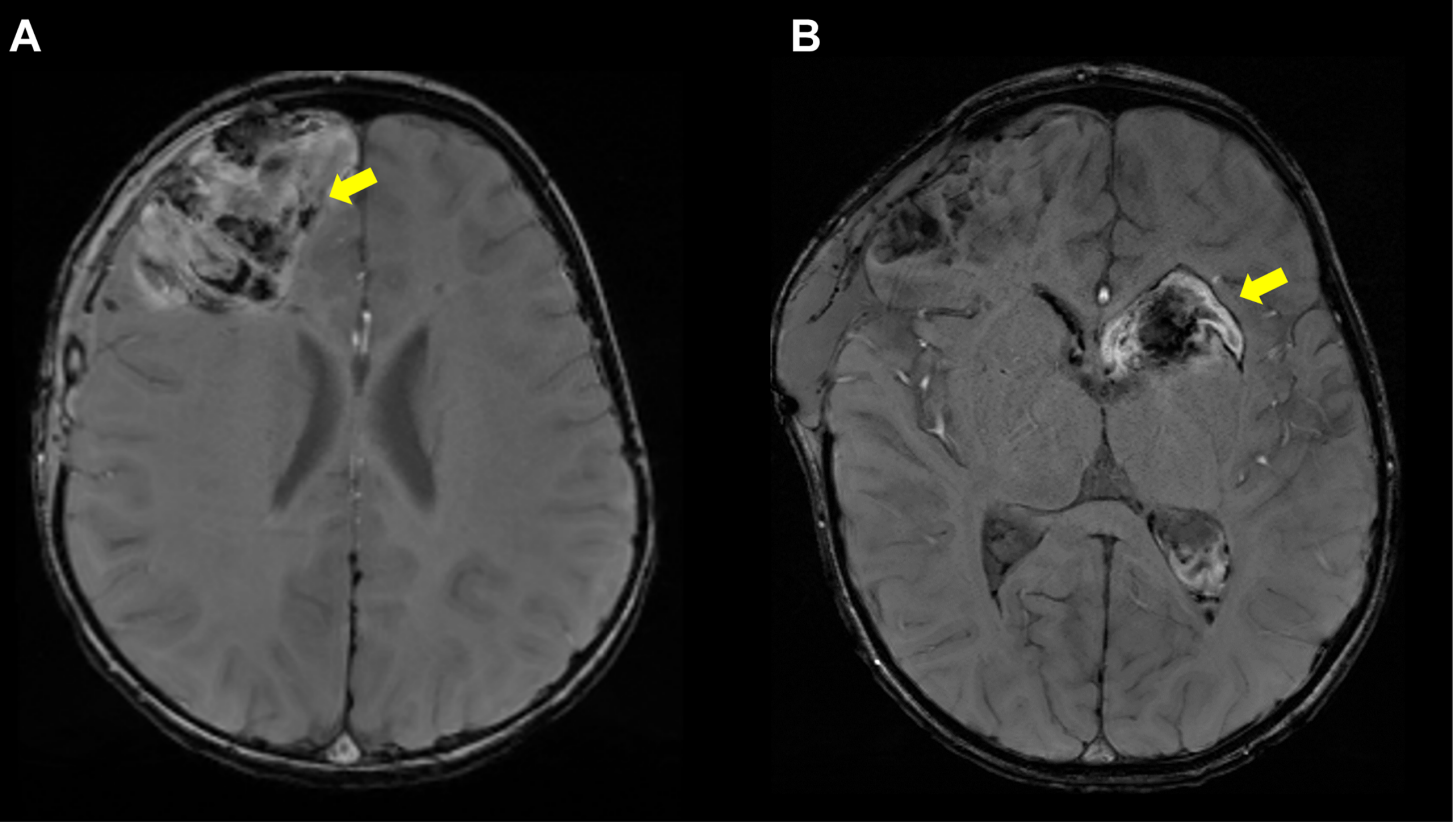
Hemorrhagic Strokes in DADA2. **(A)** Axial susceptibility weighted imaging (SWI) sequence showing right frontal intraparenchymal hemorrhage in a 2 year 5-month-old patient. **(B)** Axial SWI sequence showing left basal ganglia hemorrhage extending into the right lateral ventricle in same patient, now 2 year 6 months old. Notably, the patient was not on any anticoagulation prior to her hemorrhages.

**Figure 6:**
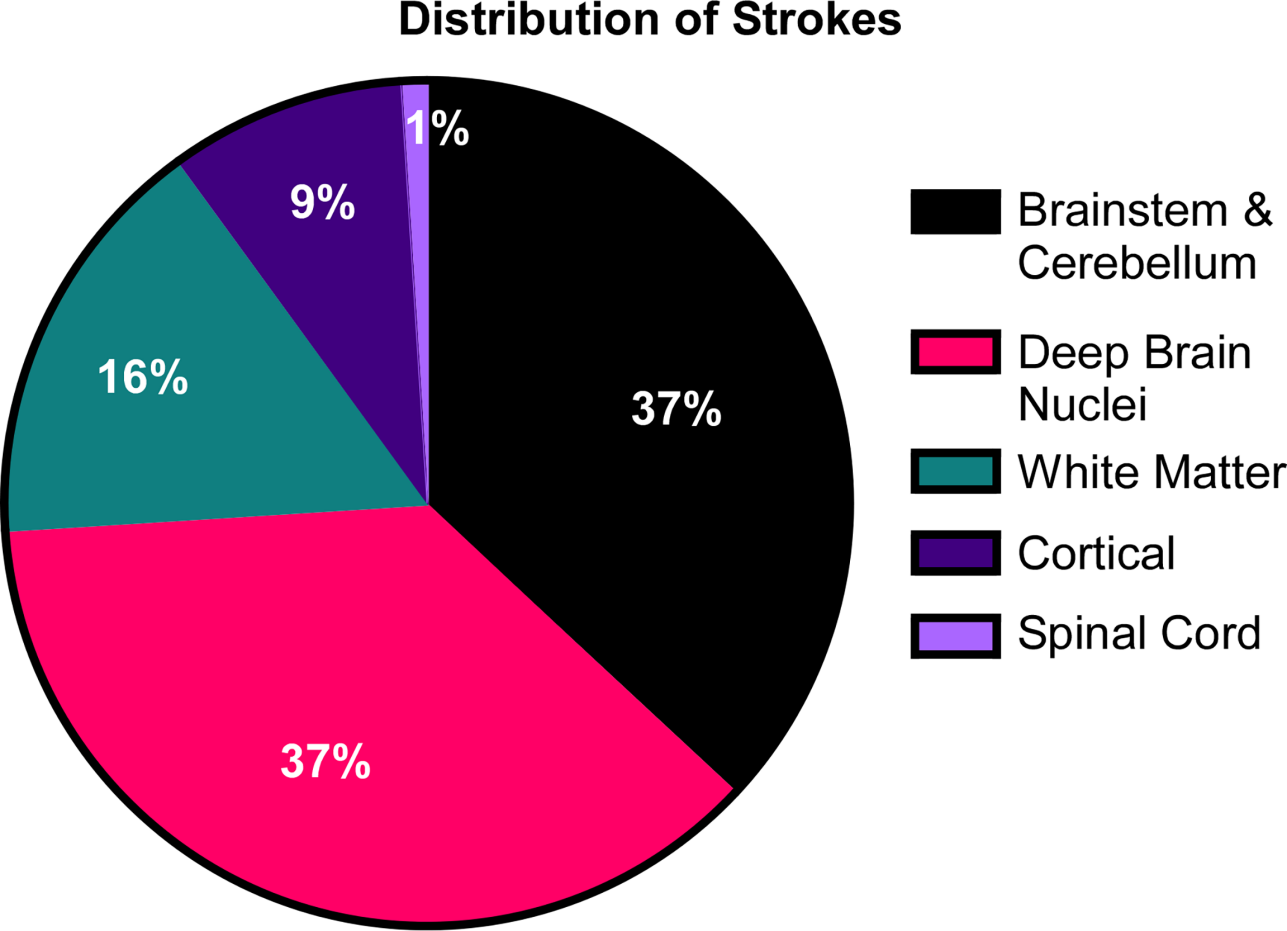
The distribution of strokes in DADA2. In the cohort of 58 patients, 25 patients amassed a total of 76 lacunar strokes. Their distribution was focused primarily in the deep brain nuclei, brainstem and cerebellum.

All patients were initially screened with MRI to detect evidence of prior strokes. Twenty-four of the 58 patients (41%) had a history and MRI confirmation of at least one stroke. One patient was found on screening MRI to have evidence of a previously unrecognized stroke that occurred sometime in his 55-year life span. Thirty-three of the 58 patients had no history and no MRI evidence of prior stroke. Most strokes occurred during episodes of inflammation, although, in some, strokes were not accompanied by notable fevers or other signs of inflammation. Ischemic strokes were observed in 24 patients with MRI showing evidence of acute or chronic small vessel ischemic infarcts (**Figure 4**). Hemorrhagic strokes were observed in 7 patients (**Figure 5**), with 6 of the 7 also demonstrating prior ischemic strokes. One patient presented with a large hemorrhagic stroke, without a history of ischemic strokes. There were a total of 76 strokes in the 25 patients, with a mean number of 3 strokes per affected patient (range 1-11). Nineteen patients had more than one stroke. They varied in location (**Figure 6**) with three quarters of the strokes occurring in the brain stem, cerebellum, and deep brain nuclei. A smaller percentage occurred in the white matter, cortex, and spinal cord. The most common sites for lacunar strokes included the thalamus, midbrain, pons, basal ganglia, and internal capsule. The average age of presentation of the first stroke was 6.5 years with a range of 0.4 to 21 years. Twelve patients presented with their first stroke before the age of 5 years.

Intrinsic coagulopathy is uncommon in patients with DADA2; thus, it was initially assumed that the hemorrhagic strokes were sequelae of the concomitant use of antiplatelet agents, warfarin, or both. However, one patient presented with hemorrhagic strokes with no prior history of ischemic stroke or the use of these medications, raising the suspicion of intrinsic endothelial dysfunction in DADA2 patients (15). Other causes of coagulopathy were ruled out. Brain biopsy obtained pre-DADA2 diagnosis in this patient revealed an organizing hematoma with associated mixed inflammatory infiltrate. Four patients manifested severe sequelae of their hemorrhagic strokes (Patients 18, 20, 37, 39).

Neuropsychological testing of 7 patients with histories of stroke and suspected abnormalities revealed moderate deficiencies. However, as neuropsychological testing was not performed on all patients, we cannot report the incidence of this finding.

Intracranial MRA was unremarkable in 18 patients. Although there were abnormalities on MRA reported in 2 patients, it should be noted that there were no compelling signs of vasculitis in any of the MRAs in our cohort, including aneurysms of the cerebral vasculature. Most importantly, the abnormalities reported did not correlate with any of the patients’ clinical findings, nor were they thought to contribute to the development of the strokes. It should be noted, however, that there are reports in the literature of intracranial aneurysms in patients with DADA2 (16). MRI revealed the presence of posterior reversible encephalopathy syndrome (PRES) in two patients with hypertension (Patients 24 and 46).

##### Other Neurologic Involvement

Involvement of the cranial nerves was common post-stroke, including ptosis secondary to cranial nerve III involvement, optic nerve or retinal involvement, anosmia confirmed by the University of Pennsylvania Smell Identification (UPSIT) standardized test secondary to cranial nerve I involvement, cranial nerve IV and VI palsies manifested by abnormal extraocular movements, and cranial nerve VII manifesting as a facial palsy.

Internuclear ophthalmoplegia reflecting brainstem involvement was seen in 2 patients. Sensorineural hearing loss, often sudden in onset, was observed in 5 patients. One patient presented with symptoms of an acute transverse myelitis after a spinal cord infarction. EMG of the lower extremities revealed a neurogenic process selectively affecting motor axons in the legs, most consistent with a lumbar expansion anterior spinal cord syndrome (Patient 18). A 14-year-old boy (Patient 10) developed a foot drop due to a pseudoaneurysm of the internal iliac artery, a complication of a bone marrow biopsy. He was treated with gabapentin for neuropathic pain and the pseudoaneurysm was embolized. EMG at the time revealed axonal sensorimotor neuropathy.

##### Vasculitis/Vasculopathy in Other Major Organs

The vascular involvement in DADA2 can extend to multiple other organs, including the liver, spleen, kidneys, heart, and bowel. Five patients experienced bowel necrosis, presumably a result of vasculitis, with subsequent gastrointestinal bleeding. Abdominal MRA revealed arteritis, aneurysms and/or stenosis of abdominal vessels in 8 patients. Four patients demonstrated defects in their spleens suggestive of prior infarcts. Renal cortical lesions suggesting a prior infarct were identified in 13 patients. Not all patients underwent abdominal MRA, so these results may underestimate the incidence of abnormalities. Significant proteinuria was seen in 4 patients and persistent hematuria in 1 patient (Patient 21). Systemic hypertension was found in 15/58 patients (26%). Many of these patients had been diagnosed with polyarteritis nodosa prior to having genetic testing.

Two patients (Patients 31 & 37) developed an idiopathic cardiomyopathy, a clinical feature described in at least one other patient in the literature (17). Coronary artery calcifications were observed in a 26-year-old (Patient 49) with no other known risk factors and may represent post-vasculitis sequelae. Abnormal EKGs were observed in 8 of 34 patients evaluated: atrial fibrillation in 2, right bundle branch block in 2, and sinus bradycardia in 4.

**Figure 7:**
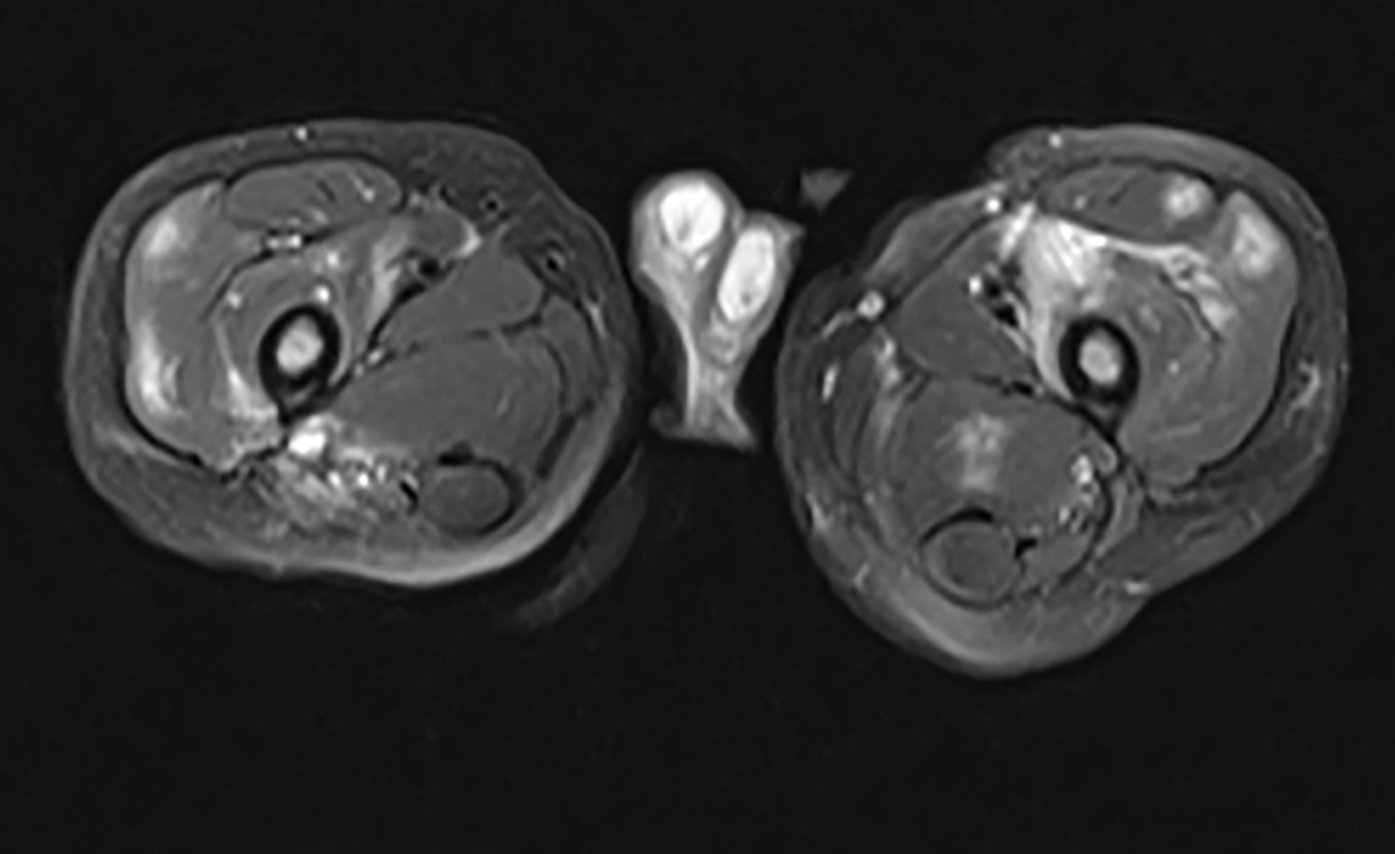
Inflammatory Myopathy in a DADA2 Patient. A 2-year-old boy presented with refusal to bear weight. He was febrile and preferred to hold his hips in a flexed position. MRI of the pelvis revealed multiple, diffuse, scattered areas of enhancement on T2 weighted imaging, bilaterally. Subsequent muscle biopsy noted multifocal perivascular inflammation as well as endomysial inflammation with both T and B lymphocytes present. There was also an increased number of eosinophils scattered throughout the biopsy specimen.

Inflammatory myositis was also observed in some of our patients. Muscle biopsies were obtained from 3 symptomatic patients (Patients 8, 14 & 39) who exhibited diffuse muscle enhancement on MRI, revealing inflammatory myositis (**Figure 7**). One additional patient (Patient 58) presented with severe muscle pain; however, no biopsy or imaging were obtained.

**Figure 8:**
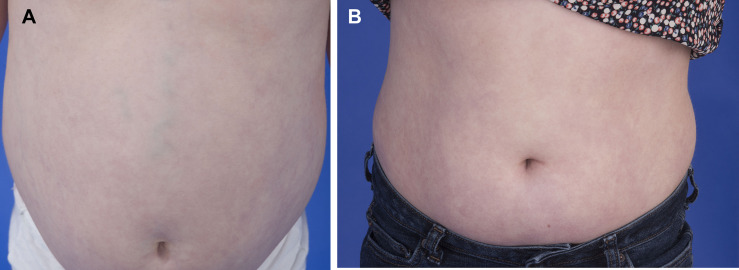
The portal hypertension in DADA2 can respond positively to anti-TNF therapy. **(A)** A 7-year-old patient with marked splenomegaly (16 cm) due to portal hypertension and variceal enlargement of abdominal vessels. **(B)** The same patient 6 years post initiation of anti-TNF therapy with reduction in spleen size (14.2 cm) and reduced vascular prominence.

The liver in DADA2 patients is thought to be subject to vascular involvement but can also have intrinsic disease as well. Hepatomegaly was diagnosed by ultrasound in 29 patients (50%) and splenomegaly in 31 patients (53%), while hepatosplenomegaly was observed in 22 patients (38%). Of the 56 patients who had transient elastography performed to quantify liver fibrosis, 14 (25%) had evidence of increased liver fibrosis, with measurements of ≥7kPa. Seven patients (12%) presented with portal hypertension. All 7 underwent liver biopsy with evidence of hepatic abnormalities appreciated in each. Liver histology revealed hepatoportal sclerosis, portal hepatitis, portal vasculopathy, and nodular/focal regenerative hyperplasia. Patient 37 with portal hypertension and focal regenerative hyperplasia required a splenorenal shunt following repeated massive variceal bleeds. The liver disease in this patient initially progressed despite treatment with a TNF inhibitor. Elevated serum ammonia levels have necessitated treatment with rifaximin and lactulose, however liver function stabilized over time. It is unlikely that anti-TNF therapy contributed to the progression of this patient’s liver disease, since there are reports that anti-TNF therapy has been beneficial in patients with hepatitis B and C-related liver disease (18) and animal models of steatohepatitis (19). Variceal enlargement of superficial abdominal vessels secondary to portal hypertension was observed in one patient (Patient 32), with noticeable improvement with anti-TNF treatment (**Figure 8**).

##### Peripheral Vasculopathy/Vasculitis

Evidence of significant peripheral vascular disease (PVD) was seen in 5 patients with symptoms ranging from numbness and paresthesia to gangrene.

**Figure 9:**
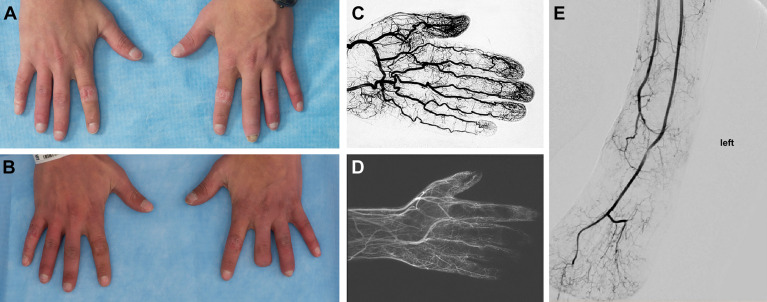
Peripheral Vascular Disease in DADA2. **(A)** 25-year-old with inflamed digits and digital resorption. **(B)** The same patient, 3 years later status post partial amputation of the left third digit following worsening peripheral vascular disease that resulted in the development of gangrene. **(C)** Angiogram of a normal hand. **(D)** Angiogram of this patient prior to the amputation, revealing severe occlusive disease of the palmar and digital arteries. The medium and small vessel involvement most closely resembles thromboangiitis obliterans. **(E)** Angiogram of a lower extremity in a patient with DADA2 showing a loss of distal vasculature perfusion.

**Figure 10:**
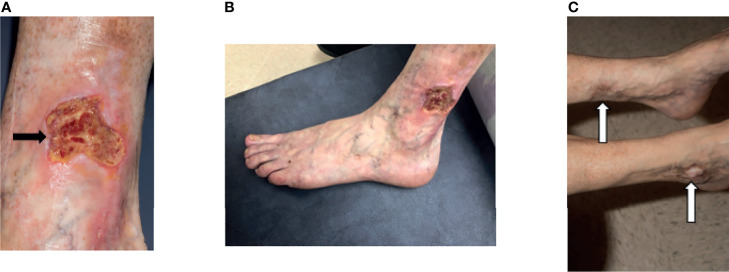
Skin ulceration in DADA2. **(A)** A 40-year-old developed a well-defined, irregularly shaped ulceration with overlying yellow fibrinous exudate (black arrow). The ulcer was not infected. There had been antecedent trauma 3 months prior with a small skin tear. This lesion developed while on adalimumab and screening for neutralizing antibodies to adalimumab was negative. **(B)** Distal to the knees there was increasing mottled, somewhat reticular erythema representing livedo racemosa. **(C)** Further examination revealed multiple hypopigmented atrophic plaques consistent with scars from prior ulcerations (white arrows).

• 28-year-old male (Patient 22) with Raynaud’s phenomenon, bone resorption, and necrotic tissue of various digits (**Figure 9**). Digital brachial index (DBI) of upper and lower extremities showed abnormal waveform and decreased pressure in several upper and lower digits. MRA demonstrated little to no blood flow to several digits. Despite treatment with anti-TNF agents, calcium channel blockers, and sildenafil, PVD continued to progress, necessitating partial amputation of several digits.
• 26-year-old female (Patient 41) with numbness and coolness of toes, with intermittent swelling and pain. DBI revealed flat waveform and no detectable pressure in her toes. There was significant small vessel disease in her toes as measured by lower extremity peripheral Pulse Volume Recording/Ankle-Brachial Index test (PVR/ABI).
• 39-year-old female (Patient 45) with a history of multiple chronic ankle ulcerations (**Figure 10**) and Raynaud’s phenomenon. Lower DBI waveform and pressure was abnormal in her digits, suggesting small-vessel disease. MRA revealed attenuated appearance of the peroneal artery.
• 14-year-old female (Patient 15) with a complaint of severe Raynaud’s phenomenon was found on MRA of her lower extremities to have a diffusely abnormal appearance to the small arterial branches in her feet, with multiple stenoses and irregularities of the small vessels.
• 11-year-old male (Patient 10) with transient pain, numbness, and paresthesia of his limbs. MRA of upper extremities revealed irregularity and occlusion of several distal arteries.

Three additional patients have noted numbness and paresthesia of their extremities and are awaiting further evaluation. Prior to the diagnosis of DADA2, two patients were diagnosed with reflex sympathetic dystrophy (RSD), a form of complex regional pain syndrome (CRPS). It is conceivable that this may be a result of microscopic vasculopathy with damage to peripheral nerves.

#### Immune Dysregulation

Diffuse adenopathy, a manifestation of lymphoproliferation, was observed in 11/58 (19%) patients. At the initial evaluation, serum immunoglobulins were abnormal in 38/58 patients (66%), with 36/58 (62%) demonstrating low IgM, 32/58 (55%) low IgG, and 25/58 (43%) low IgA (**Table 1** and **Supplemental Table 2**). Twenty-two percent of patients in this cohort received prolonged immunoglobulin replacement for hypogammaglobulinemia. Specific antibody responses to vaccination against tetanus, *Hemophilus influenzae*, rubella, diphtheria, and pneumococcal serotypes were assessed in 39 patients. Inadequate antibody levels, indicating poor memory response, were noted in 16/39 (41%) patients. One patient demonstrated inadequate response to all antigens tested (Patient 44). Of the antigens tested, the most common inadequate response was to pneumococcal antigens in 28% of all patients tested. Inadequate response to *Hemophilus influenzae* was noted in 23% of all patients tested.

##### Response to COVID-19 Infection/Immunization

Twenty eight out of 36 eligible patients received COVID-19 vaccination, and none had adverse reactions. There were seven reported COVID-19 infections. Of these seven, five were completely unvaccinated at the time of infection. Of the two remaining, one had received the first dose of vaccine one week prior to testing positive and had mild symptoms; the other had received a dose of a whole virus vaccine greater than one month prior. He developed COVID pneumonia and hemophagocytic lymphohistiocytosis and died due to complications.

##### Lymphocyte Phenotyping

Lymphocyte phenotyping of DADA2 patient peripheral blood by flow-cytometric analysis was obtained in 51 patients and was abnormal in 41 (80%) (**Table 2**). The most frequent finding was absent or low class-switched memory B cells (CD20^+^/CD27^+^/IgM^-^). There was a statistical correlation between the presence of low memory B cells and low IgG serum levels (P<0.05). Variable changes of other B cell subsets including immature/transitional B cells (CD20^+^CD10^+^ and CD20^+^/IgM^+^/CD10^+^), unswitched memory B cells (CD20^+^/CD27^+^/IgM^+^) and B cells associated with autoimmunity (CD19^+^/CD23^low^/CD38^low^) were also observed. Low levels of effector memory T cells (CD3^+^/CD4^+^/CD62L^-^/CD45RA^-^), central memory T cells (CD3^+^/CD4^+^/CD62L^+^/CD45RA^-^), and NK cells were also observed. Lymphocyte phenotyping abnormalities remained relatively constant over time.

**Table 2:** Immunophenotyping of DADA2 patients.

		Normal	Decreased	Increased
**CD3 T cells**	**T cells**			
** #**	** **	39/51	7/51	5/51
** %**	** **	**76%**	**14%**	**10%**
**CD4 T cells**	**CD4 T cells**			
** #**	** **	45/51	3/51	3/51
** %**	** **	**88%**	**6%**	**6%**
**CD8 T cells**	**CD8 T cells**			
** #**	** **	38/51	5/51	8/51
** %**	** **	**74%**	**10%**	**16%**
**CD4/CD26L^+^/CD45RA^+^**	**Naïve T cells**			
#	** **	39/48	3/48	6/48
%	** **	**81%**	**6%**	**13%**
**CD4/CD62L^-^/CD45RA^-^**	**Effector memory T cells**			
** #**	** **	22/48	26/48	0
** %**	** **	**46%**	**54%**	
**CD4/CD62L^+^CD45RA^-^**	**Central memory T cells**			
** #**	** **	30/48	16/48	2/48
** %**	** **	**63%**	**33%**	**4%**
**CD20^+^**	**B cells**			
** #**	** **	28/51	9/51	14/51
** %**	** **	**55%**	**18%**	**27%**
**CD20^+^/CD10^+^**	**Immature/ transitional B cells**			
** #**	** **	28/46	8/46	10/46
** %**	** **	**61%**	**17%**	**2%**
**CD20^+^/IgM^+^/CD10^+^**	**Immature/ transitional B cells**			
** #**	** **	28/47	8/47	11/47
** %**	** **	**60%**	**17%**	**23%**
**CD20^+^/CD38^+^/CD10^+^**	**Transitional B cells**			
** #**	** **	27/46	9/46	10/46
** %**	** **	**59%**	**20%**	**21%**
**CD20^+^/CD27^+^**	**Memory B cells**			
** #**	** **	21/47	22/47	4/47
** %**	** **	**45%**	**47%**	**8%**
**CD20^+^/CD27^+^/IgM^-^**	**Class switched memory B cells**			
** #**	** **	14/47	32/47	1/47
** %**	** **	**30%**	**68%**	**2%**
**CD20^+^/CD27^+^/IgM^+^**	**Unswitched memory B cells**			
** #**	** **	30/46	15/46	1/46
** %**	** **	**65%**	**33%**	**2%**
**CD19^+^/ CD21^low^/CD38^low^**	**Associated with autoimmunity**			
** #**	** **	35/47	9/47	3/47
** %**	** **	**74%**	**19%**	**6%**
**NK Cells**	**NK cells**			
** #**	** **	25/51	24/51	2/51
** %**	** **	**49%**	**47%**	**4%**

#Refers to number of patients with either normal, decreased, or increased lymphocyte subset divided by the number of patients tested.

%Refers to the percentage of patients tested with either normal, decreased, or increased lymphocyte subset.

#### Hematologic Abnormalities

Complete blood cell counts revealed cytopenias in 28/58 patients (48%) (**Table 1**). Within the cohort, 6/58 patients (10%) developed pancytopenia, 7/58 (12%) developed severe anemia, 9/58 (16%) immune-mediated neutropenia, 8/58 (14%) lymphopenia, and 5/58 (9%) thrombocytopenia (**Table 1**).

Bone marrow evaluation was performed in 24 patients and correlated with the laboratory findings. Six patients diagnosed with lymphopenia had hypocellular marrows. Bone marrow biopsies were obtained in 5 patients early in the course for evaluation of fever and revealed a range of hypocellular to hypercellular marrows with trilineage hematopoiesis and cellular maturation, and no evidence of blasts or malignancy.

**Figure 11:**
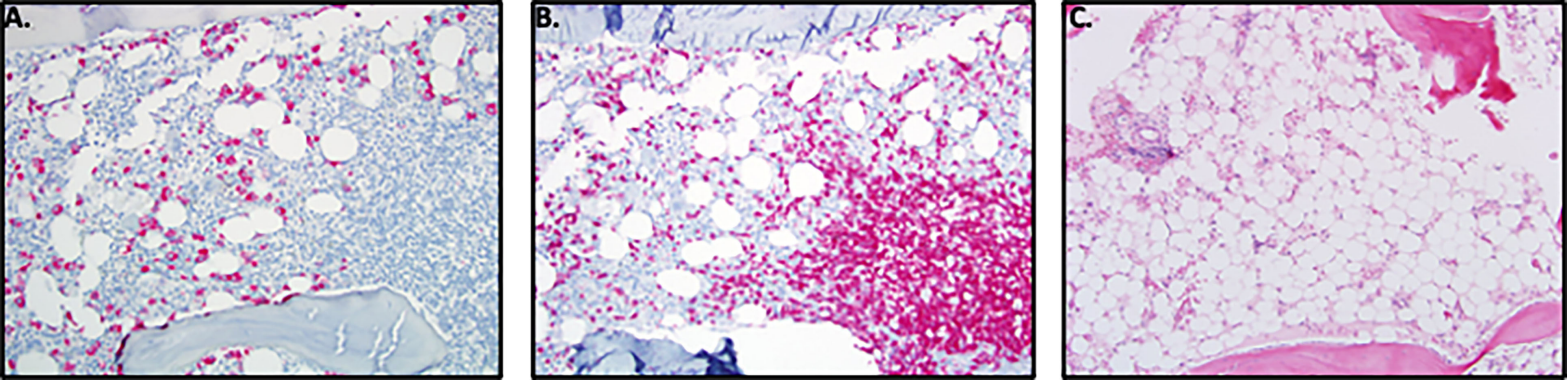
Bone Marrow Findings in DADA2. **(A)** 16-year-old male with history of DADA2 with severe neutropenia (ANC=0.02 K/uL). The marrow, when stained for MPO shows myeloid hypoplasia with presence of decreased immature myeloid precursors and absence of maturing forms consistent with maturation block or loss of myelocytes, metamyelocytes and neutrophils by immune mechanism. **(B)** Prominent T-cell aggregates and T-cell infiltrate in marrow when stained for CD3 in the marrow of the 16 y/o male with neutropenia. **(C)** 11-year-old male with progressive pancytopenia while on TNF inhibition. Bone marrow biopsy revealed marked hypocellularity for age morphologically overlapping with aplastic anemia. There was severe myeloid and erythroid hypoplasia with rare megakaryocyte clusters.

The typical bone marrow findings in patients with immune mediated neutropenia included a hypercellular marrow, with increased T-cell aggregates. (**Figures 11A, B**). In addition, bone marrow biopsy revealed evidence for myeloid hypoplasia, decreased immature myeloid precursors, and the absence of maturing forms suggesting either a maturation block or loss of myelocytes, metamyelocytes, and neutrophils by immune mechanism. Eosinophilia was also noted in several bone marrow samples. Pure red cell aplasia (PRCA) has been previously described in patients with DADA2 (20, 21) and was observed in 3 unrelated patients in this cohort (Patients 26, 28 & 31). In the other patients with severe anemia, trilineage hypoplasia and myeloid hypoplasia were noted (**Figure 11C**). Evidence of iron overload was observed in 4 patients, presumably a result of frequent red blood cell transfusions. The predominant secondary form of anemia was anemia of inflammatory disease.

#### Other Laboratory Findings

##### Inflammatory Markers

At the time of initial evaluation, erythrocyte sedimentation rate was elevated in 71% of patients and C-reactive protein was elevated in 84% of patients (**Table 1**). Serum iron was decreased in 46% of patients before the initiation of anti-TNF therapy, consistent with the anemia of inflammatory disease. These data suggest an ongoing inflammatory process, regardless of symptom presentation.

##### Autoantibodies

Low titer positive ANAs were found in about a third of patients tested. Other autoantibodies were reported infrequently: 2 patients had anti-double stranded DNA antibodies along with a positive ANA; 1 patient had positive anti-ENA, anti-double stranded DNA, and anticardiolipin antibodies in addition to a positive ANA, and 1 patient had anti-ENA antibodies in addition to a positive ANA. Positive anti-neutrophil cytoplasmic antibodies (ANCA) were only noted in 2 of 31 tested (6%), with one patient only transiently positive and the second with a low titer anti-PR3. There were no anti-platelet or anti-RBC antibodies detected in any patient.

### Treatment of DADA2 Patients

Patients presented to our clinic with a history of being prescribed a wide variety of medications. Most of these medications reflect attempts to control an undiagnosed vasculitis and included: glucocorticoids (30/58), IVIG (13/58), cyclophosphamide (9/58), methotrexate (9/58), anakinra (9/58), azathioprine (7/58), mycophenolate (6/58), etanercept (6/58), rituximab (4/58), infliximab (3/58), cyclosporine (2/58), canakinumab (2/58), alpha-interferon (1/58), colchicine (2/58) and G-CSF (2/58) dapsone (1/58), colchicine (2/58), adalimumab (1/58), tocilizumab (1/58), ATG (1/58), and G-CSF (2/58). It is important to note that glucocorticoids, although effective in reducing the inflammatory burden, did not prevent recurrence of strokes. Exposure to the TNF inhibitors included etanercept (6/58), infliximab (3/58) and adalimumab (1/58). However, anti-TNF use prior to our initiation of TNF inhibitors for DADA2 patients in 2013 was limited to two patients. Remarkably, both patients were doing well, strengthening our justification to try anti-TNF therapy in the other patients. Many patients were also taking anticoagulant/anti-platelet medications that are commonly prescribed in patients with a history of stroke. The anticoagulants included: aspirin (18/58), clopidogrel (8/58), heparin (4/58), and warfarin (2/58). A history of taking anti-coagulants or anti-platelet medications was seen in 6/7 patients with hemorrhagic strokes.

#### Anti-TNF Therapy

With the recognition that there was perivascular TNF present on DADA2 patient skin biopsies, starting in June 2013, all patients with DADA2 were offered treatment with anti-TNF agents in attempt to reduce glucocorticoid use and prevent neurologic events (22). Three patients declined. The patients received etanercept (at a dose of 0.8 to 1.2 milligram per kilogram body weight to a maximum of 50 mg weekly), adalimumab (40 mg every 1 to 2 weeks), infliximab (4 to 5 mg per kilogram every 6 weeks), or golimumab (50 mg weekly). Etanercept was the initial therapy started in the majority, primarily due to the ease of dosing in the pediatric population. Due to worsening non-neurologic disease, 4 patients were transitioned from etanercept to a monoclonal antibody against TNF, with a 5^th^ transitioning to a monoclonal antibody following the development of optic neuritis while on etanercept.

**Figure 12:**
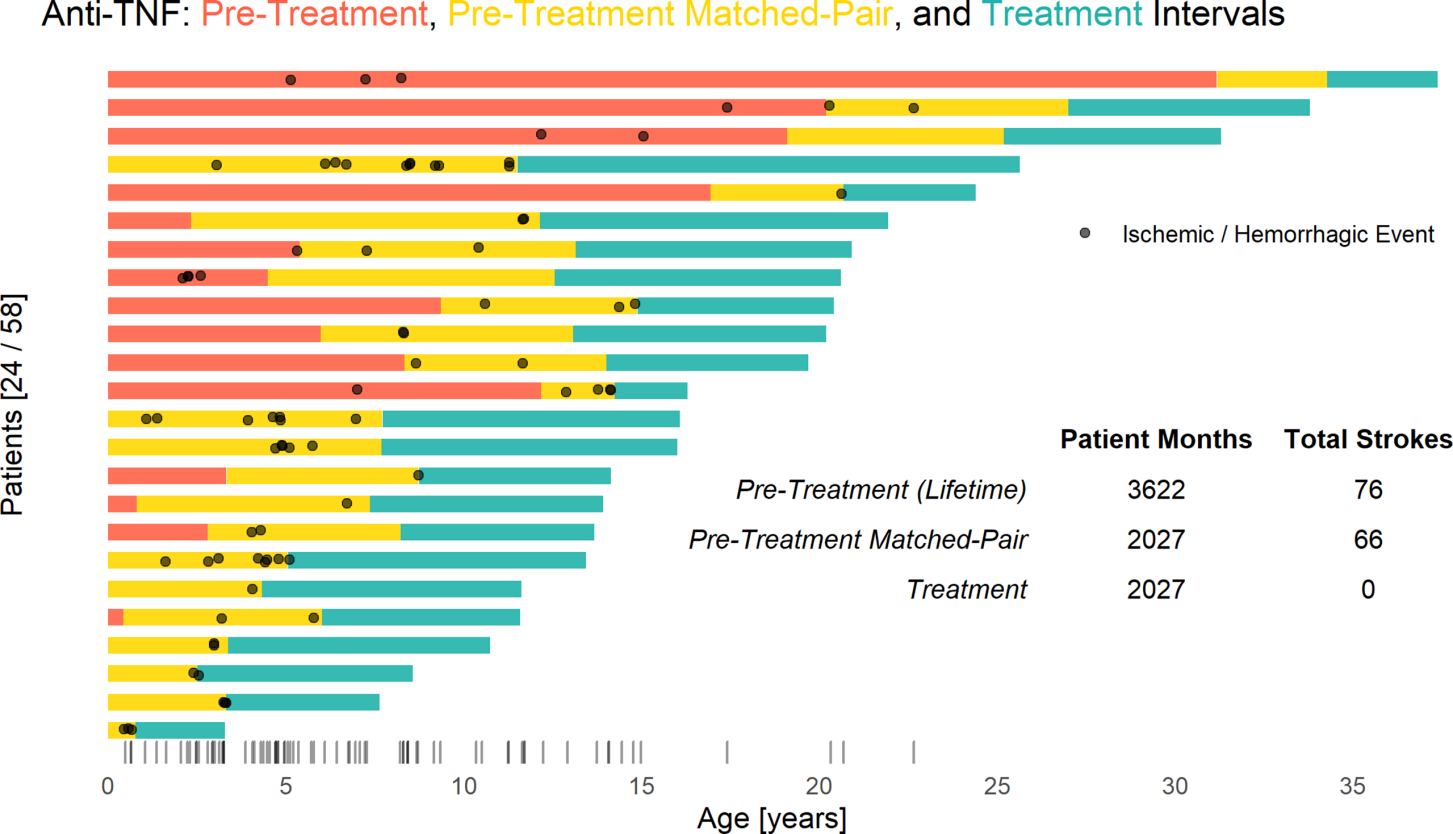
Anti-TNF treatment significantly reduces stroke risk in DADA2. Each bar represents a single patient’s respective anti-TNF treatment intervals and stroke incidence. Prior to starting TNF inhibition (red and yellow) there were a total of 76 strokes in 3,622 cumulative patient-months. No ischemic or hemorrhagic strokes have occurred in 2027 cumulative patient-months during TNF inhibition (blue), whereas 66 strokes were observed in a retrospective matched-pair (yellow) analysis (P < 0.001 by the Exact McNemar test). Overall distribution of stroke incidence by age shown *via* rug plot.

The treatment with anti-TNF agents has benefited the patients in several ways. We compared the number of individuals with or without stroke before and after treatment. Among 24 individuals with a history of one or more strokes, in 3622 patient months prior to anti-TNF therapy, there were 76 strokes reported. No strokes were observed in 2027 patient months since anti-TNF treatment was initiated, P < 0.0001 (**Figure 12**). There was one additional adult patient who was, incidentally, found to have had a prior stroke on routine screening MRI that occurred at some point in his 55 years of life. At the current time, we are aware of no other therapy, biologic or otherwise, that is as effective in preventing strokes in DADA2.

**Figure 13:**
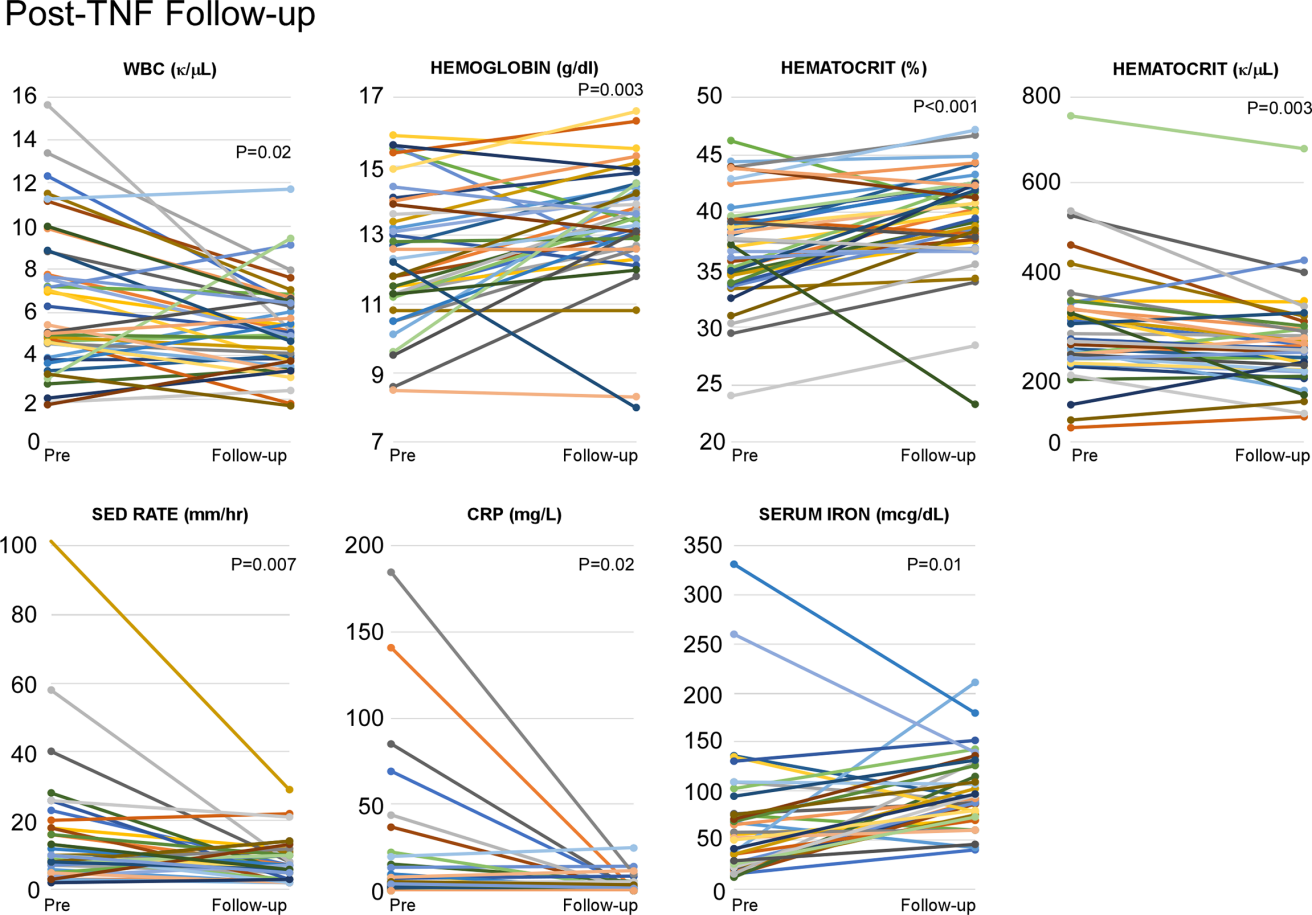
Anti-TNF agents help to reduce the inflammatory burden in DADA2 patients. Analysis of individual inflammatory burden (ESR, CRP, WBC, hemaglobin, hematocrit, platelet count, and serum iron) was completed both pre- and post-anti-TNF initiation. There were statistically significant decreases in WBC (p=0.01), platelet count (p=0.003), ESR (p=0.007), CRP (p=0.02) as well as statistically significant increases in hemoglobin (p=0.03), hematocrit (p=0.001), and serum iron (p=0.01).

In addition to the major reduction in stroke occurrence, a statistically significant decrease in inflammatory burden (ESR, CRP) followed anti-TNF treatment initiation. Also noted was an improvement in CBC parameters (WBC, hemoglobin/hematocrit, and platelet count) (**Figure 13**). Serum iron levels increased significantly as systemic inflammation decreased, while normalization of iron overload was observed in two patients (Patients 26 and 28). Previous work from our group has shown that anti-TNF therapy reduces NFkB and IFN inflammatory signatures, restores skewed M1:M2 macrophage differentiation, reduces perivascular TNF and restores endothelial integrity in individuals with vascular manifestations of DADA2 (3).

**Figure 14:**
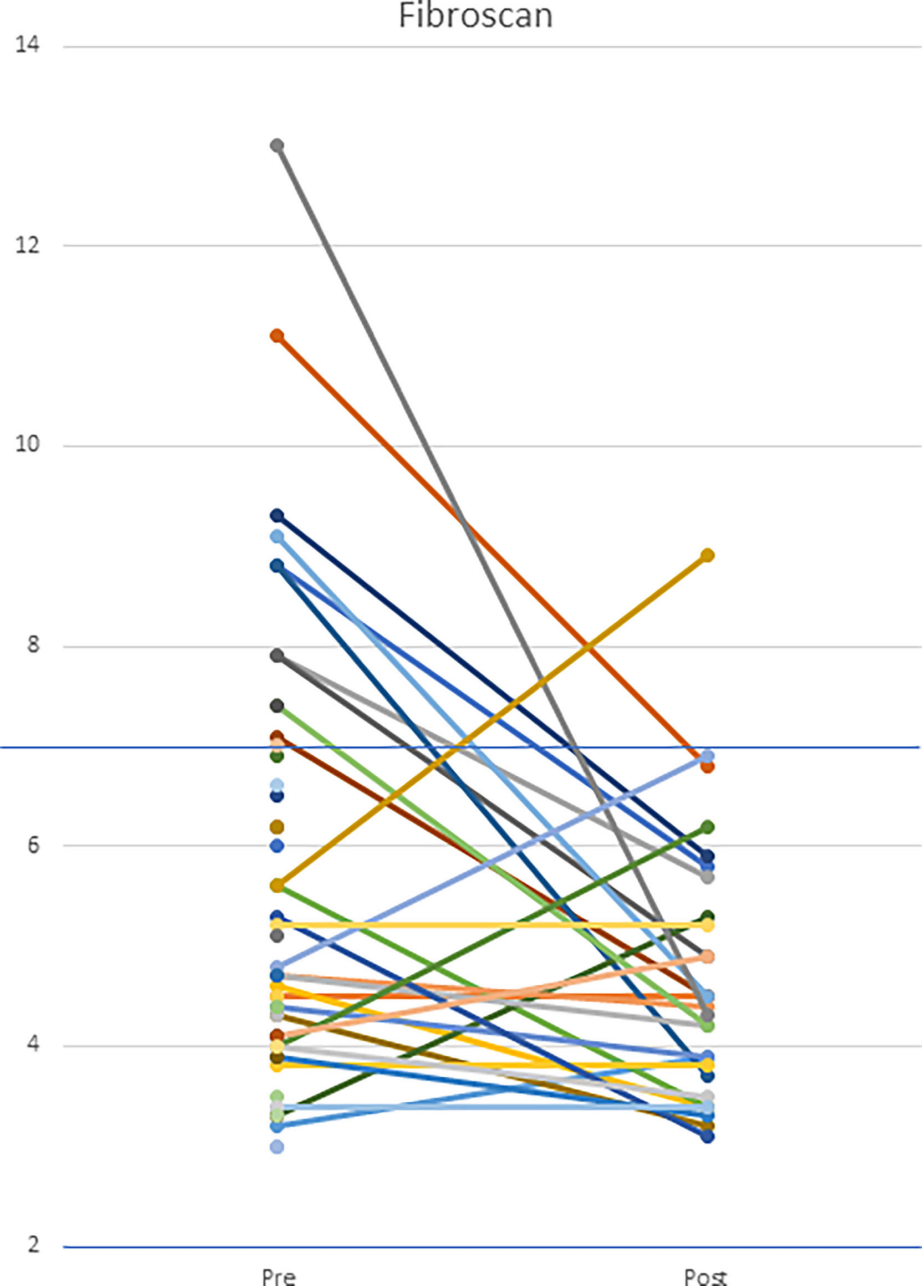
Hepatic transient elastography in patients both pre- and post-initiation of anti-TNF therapy. Patients underwent transient elastography both pre- and post-anti-TNF initiation. Upper limit of normal is defined as 7 kPa. The cohort had a statistically significant decrease in transient elastography scores (p=0.012).

Other benefits of anti-TNF treatment included improvement in the hepatic manifestations of disease, both vascular and parenchymal. Transient elastography was performed serially in 29 patients; 10/29 demonstrated evidence of hepatic stiffness on initial scans and demonstrated normal findings on follow-up. However, one patient, with initially normal values, developed evidence of hepatic stiffness on follow-up. Follow-up scans were not available for 26 patients. Of these 26, 3 had elevated levels. (**Figure 14**) Liver disease continued to progress in 1 patient (Patient 37) who had pre-existing portal hypertension and focal regenerative hyperplasia prior to the initiation of a TNF inhibitor.

The inflammatory skin manifestations of erythema nodosum and vasculitic nodules improved on treatment. There was subjective improvement of livedo racemosa in some, but not all, patients, similar to the report by Cooray et al. (23) In contrast, there was little improvement in peripheral vascular disease/Raynaud’s phenomenon, with 1 patient (Patient 22) undergoing partial digit amputations for peripheral gangrene despite treatment.

Patients with hematologic and immune dysregulation had a varied response to anti-TNF therapy with features such as neutropenia, PRCA, and hypogammaglobulinemia demonstrating the least response to TNF inhibitors. The patients with PRCA remained transfusion-dependent while on anti-TNF treatment. Trilineage bone marrow failure progressed in 1 patient with quiescent inflammatory disease while on a TNF inhibitor (Patient 39). The effect of anti-TNF treatment on serum immunoglobulin levels was variable. While some patients had a resolution of their hypogammaglobulinemia, most continued to demonstrate low serum immunoglobulin levels. Eight patients were noted to have developed hypogammaglobulinemia on follow-up while on anti-TNF treatment. One patient developed anti-adalimumab neutralizing antibodies that were detected when she presented with breakthrough fevers and panniculitis. Adalimumab was discontinued and golimumab was started along with low dose methotrexate with resolution of symptoms.

One patient (Patient 20) with advanced sequelae died at the age of 37 because of complications from trilineage bone marrow failure. She was not a candidate for HCT. Another patient with immune deficiency receiving supplemental immunoglobulin therapy, died at age 27 due to COVID-related complications. He had received one dose of the SinoPharm vaccine over one month prior to contracting COVID. It is unknown as to whether he developed antibodies to the SARS-CoV-2 spike protein. There were no other serious adverse events observed with anti-TNF therapy. One patient experienced severe joint pain on adalimumab, but subsequently did well on etanercept. Another patient developed infusion reactions to infliximab, necessitating a change to adalimumab. One patient developed features of inflammatory bowel disease, 5 years after initiation of anti-TNF therapy. Pathological findings included neutrophilic cryptitis and crypt abscess formation throughout the colon and rectum. Since anti-TNF agents are often used to treat gastrointestinal inflammatory disease, it is puzzling that inflammatory bowel disease developed in this setting. Overall, this therapy was well tolerated.

#### Hematopoietic Cell Transplantation

At the time of analysis, 6 patients had undergone a total of 10 HCTs at 3 different institutions (**Table 3**). Indications for HCT included PRCA coupled with T-cell immune-mediated neutropenia in 1, immune-mediated neutropenia in 4, and trilineage bone marrow failure in 1. Notably, one of the patients with immune-mediated neutropenia had a provisionary diagnosis of GATA2 deficiency, made before DADA2 was a recognized genetic disease (Patient 59, **Table 3**). One patient (Patient 39) had progressed from a normocellular marrow showing active maturing trilineage hematopoiesis to a markedly hypocellular marrow with trilineage failure despite being treated with anti-TNF agents for 4 years prior to bone marrow failure. The patient (Patient 26) with PRCA presented with anemia in infancy. All patients (Patients 4, 26, 34, 36) with immune-mediated neutropenia acquired the neutropenia during childhood or adolescence.

**Table 3:** Summary of patients receiving HCT.

Patient	DADA2 genotype	Transplant indication	Type of donor	Conditioning	Outcome	Complications
Patient 59 (20 yo)	G265 Stop homozygous	Presumed GATA2 with neutropenia, drug-resistant E. coli bacteremia	Carrier HLA-haploidentical sibling	Myeloablative with total body irradiation	Engrafted successfully; 5 years post transplant	Mild GVHD (skin, GI)
Patient 34 (19 yo)	H112Q/R169Q	Immune-mediated neutropenia	10/10 MUD	Reduced-intensity; no serotherapy	Engrafted day 20; graft failure day 36	Sinusoidal obstructive syndrome; engraftment syndrome
		Secondary graft failure	10/10 MUD (same donor)	Non-myeloablative; alemtuzumab serotherapy	3 donor lymphocyte infusions due to split mixed chimerism; engrafted day +19; now >2.5 years s/p 2nd transplant	Acute GVHD Grade III (skin, liver, GI); mild chronic GVHD (mouth, eye)
Patient 39 (11 yo)	R169Q/28kb deletion	Trilineage marrow failure	10/10 MUD	Reduced intensity	Engrafted day 21; >2y post transplant	GVHD (GI and skin)
Patient 36 (4yo)	L351Q/L351Q	Immune-mediated neutropenia	7/8 matched cord blood	Reduced intensity	Engrafted day 20; Graft failure day 42	Adenoviremia
		Secondary graft failure	Carrier HLA-haploidentical mother	Reduced intensity salvage regimen	Full donor chimerism day 20; now >2 y post-transplant	GVHD (GI and skin)
Patient 26 (7 yo)	H112Q/del exon 7	Pure red cell aplasia and immune-mediated neutropenia	10/10 MUD	Reduced-intensity; horse ATG serotherapy	Engrafted day 21; graft failure day 46 despite DLI	Acute hypertensive urgency, inflammatory lung infiltrates, inflammatory skin lesions, C. difficile colitis
		Secondary graft failure	10/10 MUD (same donor)	Non-myeloablative; alemtuzumab serotherapy	Engrafted neutrophils day 40; unstable/declining donor chimerism	MRSA bacteremia
		Secondary graft failure	10/10 MUD (same donor)	Non-myeloablative; rabbit ATG serotherapy	Engrafted day 20, 5 DLI's for unstable donor chimerism; >2 years s/p last transplant	Klebsiella pneumoniae lymphadenitis, cytokine release syndrome, HLH, acute GVHD Grade III (skin, liver); autoimmune hypothyroidism, hypogammaglobulinemia, avascular necrosis
Patient 4 (17 yo)	R169Q/G47W	Immune-mediated neutropenia	10/10 MUD	Reduced-intensity; horse ATG serotherapy	Engrafted day 17; now >2 years post transplant	Strep mitis bacteremia, acute GVHD (skin, grade I)

GVHD, Graft versus host disease.

MUD, HLA-matched unrelated donors.

DLI, Donor lymphocyte infusion.

All transplanted patients had been on anti-TNF agents prior to HCT, except for the patient with presumed GATA2 deficiency. Myeloablative conditioning was used in 1 patient (Patient 59) and the others received varying degrees of reduced-intensity or non-myeloablative conditioning. HLA-matched unrelated donors were used in 4 patients with a total of 7 transplantations. A fifth patient received a transplant from a haploidentical sibling. A sixth patient (Patient 36) received a transplant from a 7/8 matched cord blood and subsequently required a transplant from her HLA-haploidentical carrier mother following initial graft failure. Three patients had secondary graft failure necessitating subsequent HCTs, with 2 patients requiring 2 transplants (Patients 34 and 36) and 1 patient (Patient 26) requiring a third. These 3 patients required prolonged hospitalization ranging from 77 to more than 390 days. All 3 patients who developed graft failure had immune-mediated neutropenia as one of their HCT indications. All transplanted patients are still alive and have discontinued anti-TNF therapy. Complications of HCT included varying degrees of graft-vs-host disease in 5/6 patients. Four patients developed significant infections (**Table 3**). Patient 36 has developed a Lennox-Gastaut-like epilepsy post-transplant that has affected her neurologic progress. Notably, she did not have seizures prior to transplant and subsequent MRIs have been unremarkable for any new ischemic/hemorrhagic events.

## Discussion

This report describes the clinical features in a large cohort of patients with DADA2 from a single center. While the data from this study are extensive, there are a few salient points. In general, the clinical manifestations have been divided into the three main categories of inflammatory/vascular, hematologic, and immune dysregulation. While these divisions may be helpful in the clinical evaluation of the patient, it is important to evaluate each category as there is a tendency for patients to have clinical phenotypes that overlap the categories (**Figure 1**), or to include more than one category.

Clinical variability is further highlighted in the evaluation of family members. Genetic testing of seemingly asymptomatic family members led to 7 additional molecular diagnoses. Upon detailed evaluation, all cases had at least one clinical finding consistent with DADA2. Furthermore, in one case a sibling subsequently developed an ischemic stroke at age 21, after declining treatment with anti-TNF therapy. Similar to other monogenic disorders, DADA2 may have subclinical manifestations early in life, not becoming fully penetrant until adulthood. In some cases, the first presentation may be late yet severe and disabling, or even life-threatening (24).

These observations underscore the diagnostic limitations of this study. The NIH is a tertiary referral center, and inclusion in our cohort required a level of clinical awareness and suspicion by referring physicians. Genetic testing and enzymatic testing are complementary diagnostic techniques. *ADA2* pathogenic variants may not be detected by conventional Sanger sequencing and NGS-based genetic testing, which require the incorporation of additional diagnostic methods such as high-resolution copy-number analysis *via* multiplex ligation-dependent probe amplification (MLPA). The small structural variants, especially in the vicinity of exon 7, account for about 2-3% of pathogenic variants (25). The biochemical test is highly sensitive for the identification and/or confirmation of DADA2 patients; however, ADA2 protein activity in carriers of a monoallelic ADA2 variant is variable and may be influenced by age and infection status. A combination of enzymatic and genetic testing is advisable in the diagnosis of DADA2. Although not described to date, there remains the possibility of acquired deficiency of ADA2, either because of somatic mutations or autoantibodies.

Although the NIH clinic was initially a referral center for patients presenting with predominantly vascular phenotype, in this extended cohort less than half of the patients have presented with strokes and some patients presented as late as 20 years of age with their first stroke. Baseline screening MRI of the brain identified two “silent” strokes in patients who, by clinical history and physical examination, were expected to be free of ischemic events.

While the immunologic abnormalities in this cohort were relatively mild and similar to that described previously (26, 27), there were substantially more abnormalities observed compared to the initial observations in the first DADA2 papers (1, 2).

More than half of our patient cohort exhibited low levels of immunoglobulins at baseline, most commonly IgM and/or IgG however, some improved, perhaps as a result of treatment with anti-TNF agents. Conversely, a small number of patients developed low immunoglobulin levels as the disease progressed. Almost one quarter of patients required immunoglobulin replacement. However, severe infections were uncommon, before or after initiation of anti-TNF therapy. The majority of patients demonstrated decreased numbers of memory B and T cells in the peripheral blood as well as a decrease in NK cells (**Table 2**). In a group of 14 DADA2 patients, Schena et al. (28) observed a distribution of CD4^+^ and CD8^+^ T cells that skewed lower than healthy donors, and naïve T cells that skewed higher, but overall, most of our cohort had numbers of CD4^+^, CD8^+^, and naïve T cells that fell within the normal range. Consistent with the results of Schena, we found substantial numbers of patients with decreased memory B cells, particularly class-switched memory B cells. We did not, however, observe an increase in B cells associated with autoimmunity (CD19^+^/CD21^low^/CD38^low^). Our data are consistent with the hypothesis that there is an intrinsic B cell deficit in DADA2 (26, 28).

The hematologic manifestations seen in DADA2 can be isolated to a single cell type, such as red blood cell, lymphocytes, neutrophils, or platelets, or extend across all lineages. For some patients, hematologic abnormalities may be the most prominent manifestation of DADA2, making the diagnosis of DADA2 difficult for the unsuspecting physician. However, within our cohort, there were not any patients who solely had features of hematologic disease which should further encourage physicians seeing patients with atypical “Diamond Blackfan anemia” or “Evans syndrome” to look for other features consistent with DADA2. The cytopenias in DADA2 patients can be profound and refractory to treatment with TNF inhibitors and, unlike patients with severe immunodeficiencies, patients in our cohort specifically with neutropenia had difficulties successfully engrafting with transplantation. A recent publication detailing the outcome of HCT in 30 DADA2 patients, reported 6/30 patients experienced graft failure; however, the overall 2-year survival rate was 97% (29). The most effective conditioning regimen for HCT remains unclear although aggressive depletion of T lymphocytes is an important consideration, especially for patients with neutropenia.

Treatment with anti-TNF agents has been highly efficacious in the management of the inflammatory syndrome and vasculitis (30, 31). With time, some patients developed breakthrough symptoms that required that they be switched to one of the monoclonal TNF inhibitors. The development of neutralizing antibodies to adalimumab in one patient has prompted consideration to adding low dose methotrexate to prevent further antibody development. This paper extends the previously observed effects of anti-TNF agents by a substantially longer term and analyzes the response of a larger cohort of patients.

With the extended follow-up time, there have been other notable clinical observations. A patient with substantial portal hypertension and esophageal varices had resolution over the course of 6 years on anti-TNF therapy further emphasizing a benefit to anti-TNF therapy beyond stroke risk reduction. Conversely, a patient with a history of strokes developed total bone marrow failure over a period of 4 years while on treatment with an anti-TNF agent. This implies that close follow-up of DADA2 patients is necessary, even when the inflammatory disease manifestations are well-controlled, as phenotypic presentation may change with time and new symptoms may not be prevented with treatment. The lack of improvement in the distal vasculopathy remains a challenge but the identification of toe-brachial index testing as a potential screening tool allows for more extensive and frequent monitoring as well as the ability to identify those patients who would benefit from both core and peripheral warming. The documentation of near occlusion of both small and medium blood vessels on multiple skin biopsies may indicate a potential contributor to the development of peripheral vasculopathy. A notable clinical observation unrelated to anti-TNF treatment included an adult patient (Patient 31) with a history of transfusion dependent PRCA developing in his 50s, that spontaneously (prior to DADA2 being diagnosed) resolved with normalization of his hematocrit. The role of ADA2 on erythroid progenitors remains unclear but this “on/off” phenomenon warrants continued efforts to understand this arm of disease.

While treatment with anti-TNF agents is sufficient for most patients with a vascular phenotype, anti-TNF has little impact on severe neutropenia or pancytopenia. For the patients with these features, where transplant is not an option, investigation is ongoing with carefully chosen targeted therapies such as aggressive T-cell suppression in an attempt to improve their cytopenias. For those patients where transplant is an option, they may benefit from early hematopoietic cell transplantation so as to mitigate accumulated disease burden. Six patients in this cohort received HCTs. This has allowed for more complete analyses of their clinical presentation and response to transplant, and for comparison of their bone marrow biopsies. Patients with neutropenia tend to have hypercellular marrows with infiltrates of cytotoxic T lymphocytes, and subsequent graft failures have demonstrated decreasing myeloid chimerism with re-emergence of host lymphocytes. In the patients with graft failure, subsequent conditioning using more specific T-cell ablating agents resulted in successful engraftment.

Genetic status of related donors is important and family members who are carriers for DADA2-associated variants should be avoided, when possible (32). A carrier for DADA2 had a very low yield on bone marrow harvest and needed to undergo urgent mobilization and peripheral blood stem cell collection to augment the marrow graft (9). Whether this was a donor-specific issue or is a feature among heterozygotes of *ADA2* pathogenic mutations is unclear. As there have been several reported graft failures, as well as significant co-morbidities (often hepatic and neurologic) in HCT candidates, the best approach to HCT that optimizes engraftment while minimizing toxicity remains unclear. The optimal graft and conditioning regimens for DADA2 await further study and should certainly be a priority in future collaborative research efforts.

At the current time, gene therapy for DADA2 is not available, although research is ongoing. Zoccolillo et al. recently demonstrated success with early gene therapy studies designed to correct ADA2 expression (33). In addition, the recent report of successful treatment of ADA-SCID with ex vivo lentiviral hematopoietic stem and progenitor cell (HSPC) gene therapy, gives additional evidence that gene therapy may be adapted to treat patients with DADA2 (34).

The question of when (or if) asymptomatic patients should be treated with anti-TNF agents remains under discussion. Treatment of an asymptomatic patient adds the potential risk of immune suppression and/or the long-term concern of loss of anti-TNF efficacy from anti-drug antibodies (23). A counterargument is that until a better understanding of the risk factors for the development of strokes emerges, it is prudent to treat all patients with anti-TNF agents, unless contraindicated. In the recent comprehensive review of all published cases, Lee et al. (12) attempted to define such risk factors and demonstrated that vasculitis and strokes are typically associated with hypomorphic missense mutations that render protein activity at the low range but not completely absent in a transfection system. Mutations with a complete lack of ADA2 protein in such a transfection system are more likely to present with hematological disease. Genotype-phenotype correlations will improve as a larger number of carefully phenotyped patients are studied.

In summary, DADA2 is a heterogeneous multisystem disease with a considerable phenotypic spectrum. A recent study estimated the carrier frequency of ADA2 pathogenic variants of at least 1 in 236 individuals, corresponding to an expected disease prevalence of ~1 in 222,000 individuals and possibly 30,000 DADA2 cases world-wide (13). DADA2 should be considered in patients with early-onset fevers, rashes, and strokes, even in the absence of a positive family history (35, 36). With the expanding diversity of clinical features in patients with DADA2, it is now even more important to consider this diagnosis in patients with unexplained immunologic or hematologic abnormalities (36). It is important to promote disease awareness to physicians in different subspecialties, (especially neurology, hematology, immunology, dermatology, gastroenterology, and hepatology), who can make the diagnosis in a timely manner. Treatment with anti-TNF agents should be strongly considered for all as they significantly reduce a patient’s risk for stroke and other complications of the disease (22). Antiplatelet agents and anticoagulants are generally contraindicated, as they may increase the risk of hemorrhagic strokes. Follow-up monitoring for the appearance of new manifestations is important as additional organ system involvement can develop despite treatment with anti-TNF agents. For those with disease refractory to anti-TNF agents, HCT is potentially curative, although careful consideration should be taken as a graft failure is possible, necessitating additional transplants.

Future studies on the molecular mechanisms of the ADA2 deficiency are necessary to better understand factors that influence clinical variability, disease progression and the use of individualized therapies for patients with DADA2.

## Data Availability Statement

The original contributions presented in the study are included in the article/**Supplementary Material**. Further inquiries can be directed to the corresponding author.

## Ethics Statement

The studies involving human participants were reviewed and approved by the NIH Institutional Review Board. Written informed consent to participate in this study was provided by the participants’ legal guardian/next of kin.

## Author Contributions

KB, IA, ND, DLK, and AO conceptualized the study, collected and analyzed the data and drafted the manuscript. DS, PH, AJ, TR, and MN participated in patient recruitment and in sample collection. AS, JB, CT, CC, MM, MB, JK, DD, KC, HA, DK, GB-Y, DP, LH, AB, TH, and EC evaluated patients and contributed to data collection and analysis. RV-L, EM, OS, NM, and SR compiled and analyzed the data. All authors contributed to the article and approved the submitted version.

## Conflict of Interest

The authors declare that the research was conducted in the absence of any commercial or financial relationships that could be construed as a potential conflict of interest.

The handling editor declared a past co-authorship with several of the authors IA, DLK, and AO.

## Publisher’s Note

All claims expressed in this article are solely those of the authors and do not necessarily represent those of their affiliated organizations, or those of the publisher, the editors and the reviewers. Any product that may be evaluated in this article, or claim that may be made by its manufacturer, is not guaranteed or endorsed by the publisher.

## Supplementary Material

Supplementary Table 1 Classification of variants reported in this cohort using American College of Medical Genetics (ACMG) criteria.

Supplementary Table 2 Comprehensive immune phenotyping of DADA2 patients.

## Abbreviations

ADC, apparent diffusion coefficient; ALPS, autoimmune lymphoproliferative disease; CBC, complete blood count; CNV, copy number variant; CRP, C-reactive protein; CRPS, complex regional pain syndrome; DADA2, deficiency of adenosine deaminase 2; DBI, digital brachial index; DWI, diffusion weighted imaging; ESR, erythrocyte sedimentation rate; HCT, hematopoietic cell transplant; IFN, interferon; LDGs, low-density granulocytes; MRA, magnetic resonance angiography; MRI, magnetic resonance imaging; NET, neutrophil extracellular trap; PRCA, pure red cell aplasia; PVD, peripheral vascular disease; PVR/ABI, pulse volume recovery/ankle-brachial index; RSD, reflex sympathetic dystrophy; SWI, susceptibility weighted imaging; TNF, tumor necrosis factor; UPSIT, University of Pennsylvania Smell Identification Standardized Test.
