# Supplementary material for: The Spectrum of the Deficiency of Adenosine Deaminase 2: An Observational Analysis of a 60 Patient Cohort
Source: Front Immunol. 2022 Jan 10;12:811473. doi: 10.3389/fimmu.2021.811473 (PMC8790931; doi:10.3389/fimmu.2021.811473)
Supplement: Supplementary Table 1 — Classification of variants reported in this cohort using American College of Medical Genetics (ACMG) criteria. [file Table_1.docx]

| Variant | Genomic location | Category | Applied Criteria | Patients with variant |
| --- | --- | --- | --- | --- |
| c.-47+2T>C | chr22:17700244A>G | P | PVS1, PS3, PM2, PM3 | 15*, 16*, 17* |
| Met1Thr | chr22:17690566A>G | P | PVS1, PS3, PM2 | 20 |
| Arg34Trp | chr22:17690468G>A | P | PS3, PM2, PM3, PP1, PP2 | 23, 34 |
| Ile93Thr | chr22:17690290A>G | LP | PS3, PM2, PM3, PP3 | 20 |
| Gly47Ala | chr22:17690429C>T | P | PS3, PM2, PM3, PM5, PP1, PP4 | 21+, 22, 32, 37, 47, 48 |
| Gly47Arg | chr22:17690429C>T | P | PS3, PP1, PM5 | 1+ 8, 9, 13, 19+, 24*, 25*, 43+, 49+, 52+ |
| Gly47Trp | chr22:17690429C>A | P | PS3, PM1, PM2, PM5, PP3, PP5 | 4*, 5*, |
| Arg49fs | chr22:17209534-17209540 | P | PVS1, PM2, PM3, PP5 | 46 |
| Lys55del | chr22:17690402CCTT>C | P | PS3, PM2, PM3, PM4, PP1, PP2, | 10*, 11* |
| His91Ile*fs12 | chr22: 17690297del | P | PVS1, PM2, PP5 | 55 |
| Ala109Asp | chr22:17688177G>T | P | PS3, PM2, PM3, PP3, PP5 | 18 |
| His112Gln | chr22:17688167G>C | P | PS3, PM2, PM3, PP3, PP5, PP1 | 26, 34*, 35*, 37, 45 |
| Thr129Pro | chr22:17688118T>G | P | PS3, PM3, PM2, PP3, PP5, PP1 | 3, 8*, 9* |
| Arg169Gln | chr22:17687997C>T | P | PS3, PM2, PP1, PP5 | 2, 4*, 5*, 6+, 7, 22, 34*, 35*, 39, 40+, 42, 47, 53*+, 54*+, 55, 58 |
| Phe178Ser | chr22:17687970A>G | LP | PS3, PM2, PP3, PP5 | 44+ |
| Leu188Pro | chr22:17684643A>G | LP | PS3, PM2, PP3, PP1 | 30*, 31* |
| Trp204Cys | chr22:17684594 C>A | LP | PS3, PM2, PM3, PP3 | 7, 57+ |
| Glu244Ala | chr22:17684475T>G | LP | PS3, PM2, PP3 | 38 |
| Pro251Leu | chr22: 17684454G>A | P | PS3, PM2, PP2 | 46 |
| Ser265* | chr22: 17672660G>C | LP | PVS1, PM2 | 59+ |
| c.973-2A>G | chr22:17669339T>C | P | PVS1, PS3, PM2, PM3, PP5 | 12, 48, 50*, 51* |
| Arg306* | chr22:17670888G>A | P | PVS1, PS3, PM2, PM3, PM2, PP5 | 33 |
| Arg312* | chr22:17670870G>A | P | PVS1, PS3, PM2, PM3 | 50*, 51* |
| Del exon 7 | chr22:17669229_17669337del | P | PVS1, PS3, PM2, PM3 | 26 |
| Dup exon 7 | chr22:17669229_17669337dup | LP | PS3, PM2, PP1 | 27*+, 28*+, 29*+ |
| Glu328Lys | chr22:17669328C>T | P | PS3, PM1, PM2, PM5, PP3, PP5 | 41 |
| Leu351Gln | chr22:17669258A>T | LP | PS3, PM2, PP3, PP5 | 36+ |
| Phe355Leu | chr22:17669245G>T | P | PS3, PM1, PM2, PM3, PP5 | 41 |
| Ala357Thr | chr22:17669241 C>T | P | PS3, PM2, PM3, PP3, PP5, PP1 | 14, 42 |
| Gly358Arg | chr22:17669238C>T | P | PS3, PM2, PM3, PP3, PP5, PP1 | 14, 24*, 25*, 56+ |
| Thr360Ala | chr22:17669232T>C | P | PS3, PM1, PM2, PP3, PP5 | 23, 58 |
| Asn370Lys | chr22:17663623G>T | P | PS3, PM2, PP3, PP5, PP2, PP1 | 10*, 11* |
| Gly383Ser | chr22:17663586C>T | LP | PS3, PM1, PM2, PP3, PP5 | 33 |
| Pro425Ala | chr22:17662879 G>C | LP | PS3, PM2, PP3 | 38 |
| Pro435Ala | chr22:17662849G>C | LP | PS3, PM3, PP3, BS1, BP6 | 13 |
| Tyr453Cys | chr22:17662794T>C | P | PS3, PM2, PP3, PP2, PP5 | 2, 15*, 16*, 17*, 18, 32, |
| Val458Asp | chr22:17662779A>T | P | PS3, PM2, PM3, PP1, PP5 | 12 |
| Trp501Arg | chr22:17662408A>G | LP | PS3, PM2, PP3, PP2, PP1 | 30*, 31* |
| 13.5kb del (Exon 8-10) | chr22:17173307-17186725del | P | PVS1, PS3, PM2 | 3 |
| 28kb deletion | chr22:17695127-17723376del | P | PVS1, PM2, PM3 | 39 |

*siblings

+ homozygous
